# Contribution of Segment 3 to the Acquisition of Virulence in Contemporary H9N2 Avian Influenza Viruses

**DOI:** 10.1128/JVI.01173-20

**Published:** 2020-09-29

**Authors:** Anabel L. Clements, Joshua E. Sealy, Thomas P. Peacock, Jean-Remy Sadeyen, Saira Hussain, Samantha J. Lycett, Holly Shelton, Paul Digard, Munir Iqbal

**Affiliations:** a The Pirbright Institute, Pirbright, Woking, United Kingdom; b The Roslin Institute and Royal (Dick) School of Veterinary Studies, University of Edinburgh, Edinburgh, United Kingdom; c Department of Infectious Diseases, Imperial College London, United Kingdom; St. Jude Children’s Research Hospital

**Keywords:** avian influenza viruses, chicken, H9N2, increased virulence, PA polymerase gene mutation, poultry

## Abstract

H9N2 avian influenza viruses (AIVs) circulate in poultry throughout much of Asia, the Middle East, and Africa. These viruses cause huge economic damage to poultry production systems and pose a zoonotic threat both in their own right and in the generation of novel zoonotic viruses, for example, H7N9. In recent years, it has been observed that H9N2 viruses have further adapted to gallinaceous poultry, becoming more highly transmissible and causing higher morbidity and mortality. Here, we investigate the molecular basis for this increased virulence, comparing a virus from the 1990s and a contemporary field strain. The modern virus replicated to higher titers in various systems, and this difference mapped to a single amino acid polymorphism at position 26 of the endonuclease domain shared by the PA and PA-X proteins. This change was responsible for increased replication and higher morbidity and mortality rates along with extended tissue tropism seen in chickens. Although the PA K26E change correlated with increased host cell shutoff activity of the PA-X protein *in vitro*, it could not be overridden by frameshift site mutations that block PA-X expression and therefore increased PA-X activity could not explain the differences in replication phenotype. Instead, this indicates that these differences are due to subtle effects on PA function. This work gives insight into the ongoing evolution and poultry adaptation of H9N2 and other avian influenza viruses and helps us understand the striking morbidity and mortality rates in the field, as well as the rapidly expanding geographical range seen in these viruses.

**IMPORTANCE** Avian influenza viruses, such as H9N2, cause huge economic damage to poultry production worldwide and are additionally considered potential pandemic threats. Understanding how these viruses evolve in their natural hosts is key to effective control strategies. In the Middle East and South Asia, an older H9N2 virus strain has been replaced by a new reassortant strain with greater fitness. Here, we take representative viruses and investigate the genetic basis for this “fitness.” A single mutation in the virus was responsible for greater fitness, enabling high growth of the contemporary H9N2 virus in cells, as well as in chickens. The genetic mutation that modulates this change is within the viral PA protein, a part of the virus polymerase gene that contributes to viral replication as well as to virus accessory functions—however, we find that the fitness effect is specifically due to changes in the protein polymerase activity.

## Abstract (precis)

Avian influenza viruses, such as H9N2, cause huge economic damage to poultry production worldwide and are additionally considered potential pandemic threats. Understanding how these viruses evolve in their natural hosts is key to effective control strategies. In the Middle East and South Asia, an older H9N2 virus strain has been replaced by a new reassortant strain with greater fitness. Here, we take representative viruses and investigate the genetic basis for this “fitness.” A single mutation in the virus was responsible for greater fitness, enabling high growth of the contemporary H9N2 virus in cells, as well as in chickens. The genetic mutation that modulates this change is within the viral PA protein, a part of the virus polymerase gene that contributes to viral replication as well as to virus accessory functions—however, we find that the fitness effect is specifically due to changes in the protein polymerase activity.

## INTRODUCTION

Influenza A viruses possess a segmented, negative-sense RNA genome that is transcribed and replicated by a tripartite RNA-dependent RNA polymerase (RdRp) composed of the subunits PB2, PB1, and PA. Due to the segmented nature of its genome, influenza viruses can readily swap genes when two virus strains coinfect a single cell, in a process known as reassortment. Reassortment can result in generation of viruses with increased (1) or reduced viral fitness (2).

H9N2 avian influenza viruses (AIVs) are low-pathogenicity avian influenza (LPAI) viruses that are enzootic in poultry in many countries across Asia, Africa, and the Middle East (3–6). In afflicted countries they cause a constant burden on poultry production systems through mortality, often associated with coinfection, or through morbidity that leads to reduced egg production and bird growth rates (7–9). They also pose a zoonotic risk, as evidenced by over 60 confirmed cases of human infection, with over half of those occurring since 2015 (6).

Due to their extensive geographical range, H9N2 AIVs often cocirculate with other AIV subtypes resulting in frequent reassortment events (10). Several viruses have emerged in recent years that contain the internal gene cassette derived from H9N2 AIVs, including an avian-origin H7N9 virus which has caused human infections in China. H7N9 possesses the polymerase genes from an enzootic cocirculating H9N2 strain (11, 12). Novel genotypes of H9N2 AIV have also emerged in poultry due to cocirculation and reassortment with local highly pathogenic avian influenza virus strains; we have previously described G1-lineage H9N2 viruses in Pakistan that possess the NS gene segments from H7N3 or H5N1 strains and the polymerase genes from other Indian/Middle East lineage H9N2 viruses (13). These reassortants have replaced previously circulating genotypes of the G1-lineage H9N2 AIVs, are now the predominant genotype across the Indian subcontinent and Middle East, and display enhanced morbidity and mortality in the field (14–16).

The molecular basis for the increased pathogenicity of contemporary reassortant H9N2 AIVs has yet to be established. Thus, we set out to understand which genes are responsible for the enhanced virulence of these H9N2 viruses in poultry. We created a panel of reverse genetics (RG) reassortants between a pair of G1-lineage viruses, namely A/guinea fowl/Hong Kong/WF10/1999 (WF10), a virus representing G1-lineage viruses circulating in the late 1990s, and A/chicken/Pakistan/UDL-01/2008 (UDL-01), representative of novel G1-lineage reassortant H9N2 viruses. UDL-01 contains the HA, NA, NP, and M genes related to previously circulating enzootic G1-lineage H9N2 viruses in the region, the polymerase gene cassette and the NS gene segments from a high-pathogenicity AIV (HPAIV) H7N3 strain (13).

In this study, we found that the contemporary H9N2 virus, UDL-01, showed an enhanced replication phenotype *in vitro* compared to that of the ancestral H9N2 WF10 virus. This phenotypic difference mapped to a single amino acid residue in the PA endonuclease domain (position 26) within segment 3. This single residue also determined the replicative fitness and virulence of the virus *in vivo* and was further shown to modulate the activity of PA in a PA-X-independent manner.

## RESULTS

### Differences in plaque phenotype between two H9N2 AIV strains maps to the N-terminal half of segment 3.

**FIG 1:**
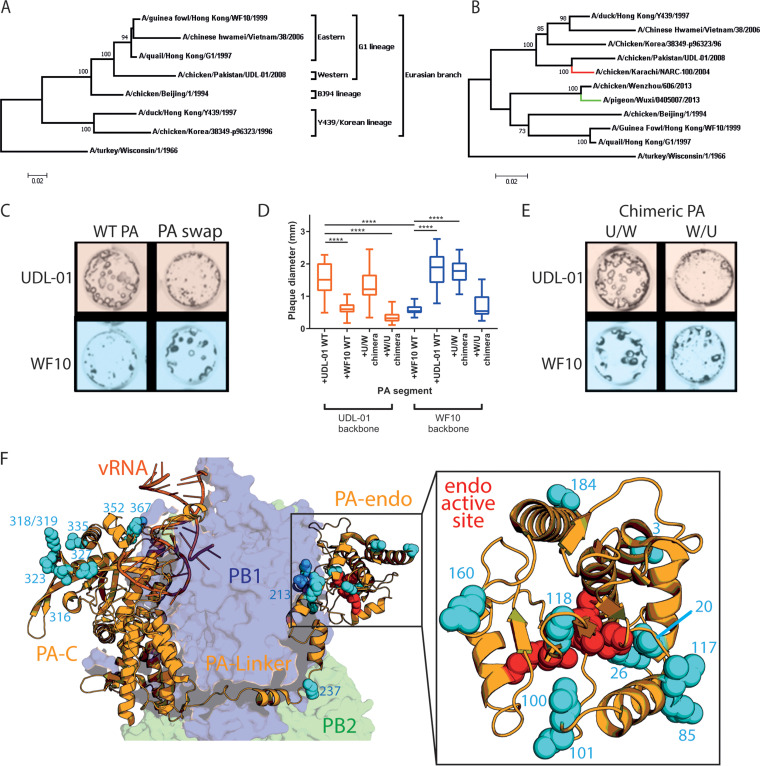
The small-plaque phenotype of WF10 maps to the N terminus of segment 3. Phylogenetic trees of hemagglutinin (A) and PA (B) were constructed to highlight the evolutionary relationship between H9N2 viruses UDL-01 and WF10. Sequences included for comparison are from lineage-defining viruses of the H9N2 subtype; sequences for H7N3 (red branch, A/chicken/Pakistan/NARC-100/2004) and H7N9 (green branch, A/pigeon/Wuxi/0405007/2013) were included to highlight past reassortment. The maximum likelihood method was used in tree generation with 1,000 bootstrap replicates in MEGA7. All nucleotide sequences were downloaded from the NCBI database. Titers of the indicated viruses were determined in MDCK cells via plaque assay under a 0.6% agarose overlay and at 72 h postinfection (h p.i.), fixed, and stained for NP using immunofluorescence. (C and E) Representative images of plaque sizes of UDL-01 and WF10 RG viruses. (D) Diameter of 20 plaques/virus measured using ImageJ analysis software. The graphs represent the average plaque diameter ± standard deviation (SD). (D) Kruskal-Wallis with Dunn’s multiple-comparison test was used to determine the statistical differences between the plaque sizes. ****, *P* < 0.0001 (F) Structure of the trimeric polymerase with viral RNA (vRNA) (dark orange) with PB2 (green), PB1 (blue), and PA (light orange). N-terminal half PA differences between WF10 and UDL-01 shown in cyan, zoomed-in PA endonuclease domain included in right panel, and PA endonuclease active set residues H41, E80, D108, E119, and K134 shown in red (PDB identifier 4WSB) (27).

We generated a panel of reciprocal reassortant viruses between the full reverse genetics systems of WF10, a virus representing G1-lineage H9N2 AIVs that circulated in the late 1990s, and UDL-01, representative of a novel reassortant G1-lineage H9N2 with genes from several previously enzootic G1-lineage H9N2 viruses and HPAIV H7N3 viruses (see Fig. 1A and B for phylogenetic trees of HA and PA genes). Wild-type (WT) WF10 virus generated small hazy plaques in MDCK cells, whereas WT UDL-01 generated significantly larger, clearer plaques (Fig. 1C and D). We tested the plaque phenotype of all reassortants and identified segment 3 as capable of reciprocating plaque phenotype between WF10 and UDL-01 (Fig. 1C and D; data for non-segment 3 reassortants not shown). UDL-01 virus containing segment 3 of WF10 presented significantly smaller plaques, while reassortant WF10 presented significantly larger plaques, relative to WT viruses (Fig. 1C and D).

To identify the region of segment 3 responsible for this alteration, two chimeric segments 3s were generated by Gibson assembly: one chimera encoded the N terminus (amino acids 1 to 367) from PA of UDL-01 and the C terminus (amino acids 368 to 716) of WF10 (U/W), while the other was vice versa (W/U). These chimeric segments were rescued by reverse genetics in the background of UDL-01 and WF10 viruses. Determination of the plaque phenotypes showed that, regardless of the rest of the virus genes, viruses containing a UDL-01 PA N-terminal coding region had significantly larger plaques that those without (Fig. 1D and E). Furthermore, UDL-01 virus, which typically presents a large-plaque phenotype, presented significantly smaller plaques when given a WF10 N terminus (Fig. 1D and E). These results show that the small-plaque phenotype could be mapped specifically to the N-terminal half of WF10 PA.

### Amino acid residue 26 in PA modulates plaque phenotype.

PA is composed of two major domains, an N-terminal endonuclease (endo) domain and a C-terminal domain (PA-C) connected by a linker region (17). The PA endo domain is a flexible appendage that hangs away from the catalytic core of the RdRp and is involved in cleaving host capped RNAs to be fed into the RdRp active site to be used as primers for viral transcription. The PA-C domain is packed close to PB1 and makes up part of the catalytic core of the viral RdRp (18).

The first 191 amino acids of PA, incorporating the endo domain, are also shared with the accessory protein PA-X. PA-X is expressed due to a ribosomal frameshift site in PA, during segment 3 translation a small proportion of ribosomes, when encountering a rare tRNA codon slip into the +2 open reading frame (ORF) of segment 3 and express a fusion protein comprised of the PA endo domain and an X-ORF from the +2 reading frame (19, 20). PA-X dampens the innate immune response though its host cell shutoff activity, mediated through degradation of cellular mRNAs and disruption of mRNA processing machinery (19, 21). The evidence for PA-X playing a role as a virulence factor in avian influenza viruses is unclear, with several studies showing either attenuation or promotion of virulence *in vivo* (22–26).

To identify amino acid substitutions responsible for modulating virus plaque phenotype, we compared amino acids in the N-terminal half of PA between UDL-01 and WF10, as well as between the X-ORFs of PA-X. We identified a total of 20 amino acid differences between PA and 2 unique to the X-ORF (Table 1). Mapping the residues onto the crystal structure of influenza PA within the context of the polymerase trimer bound to viral RNA (vRNA) showed that they lay in the PA-endo domain, the linker region, and at the N terminus of the PA-C domain (27) (Fig. 1F, Table 1). This enabled us to speculate whether any of the substitutions had a direct effect due to their proximity to known functional regions. Substitution I118T is specifically located within the endonuclease active site, while A20T, E26K, and I100V/D101E lie proximal to the active site and could potentially interfere with endonuclease activity. Therefore, these mutations, alongside the X-ORF polymorphisms and several other mutants at residues shown previously to modulate polymerase activity (28), were selected for further testing.

**TABLE 1:** Amino acid differences between the N terminus of progenitor (WF10) and reassortant (UDL-01) H9N2 segment 3 products

Amino acid position	WF10	UDL-01	Domain
3	D	N	Endo
20	T	A	Endo
26	K	E	Endo
85	A	T	Endo
86	M	L	Endo
100	V	I	Endo
101	D	E	Endo
118	T	I	Endo
160	D	E	Endo
184	S	N	Endo
213	R	K	Linker
237	K	E	Linker
316	D	G	PA-C
318	R	K	PA-C
319	E	D	PA-C
323	I	V	PA-C
327	E	K	PA-C
335	I	L	PA-C
352	D	E	PA-C
367	M	K	PA-C
X-221	L	R	X-ORF (PA-X)
X-250	R	Q	X-ORF (PA-X)

**FIG 2:**
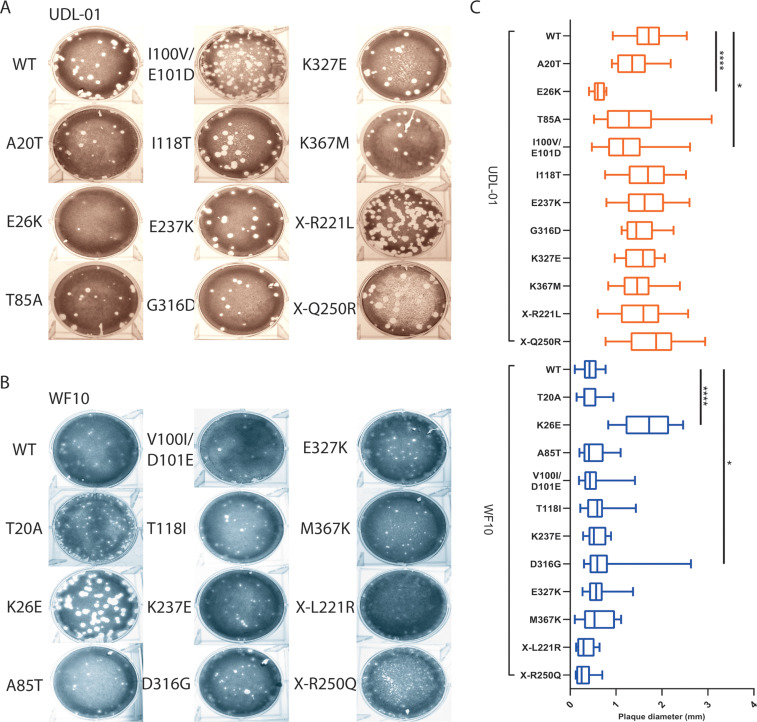
The small-plaque phenotype of WF10 maps to PA position 26. H9N2 AIVs with either a UDL-01 or WF10 backbone were rescued via reverse genetics. The plaque phenotypes of the rescues were assessed via plaque assay on MDCK cells under a 0.6% agarose overlay. After 72 h, cells were fixed and stained with 0.1% crystal violet solution and plaques imaged. (A) Visual representation of UDL-01 virus panel containing PA mutations to make them WF10-like. (B) Visual representation of WF10 virus panel containing PA mutations to make them UDL-01 like. (C) All plaque assays were performed on the same days, and the plaque size diameter of randomly selected twenty plaques for each virus was measured using ImageJ analysis software and the average plaque diameter calculated. Graph represents the average ± SD. ****, *P* < 0.0001; **, *P* < 0.0039 (Kruskal-Wallis with Dunn’s multiple comparisons).

A panel of viruses was made carrying reciprocal single amino acid substitutions at the sites identified and viruses were rescued using reverse genetics. Within the UDL-01 panel of PA mutant viruses, the following two mutants gave significantly smaller plaque sizes than UDL-01 WT: the E26K mutant produced comparably sized plaques to that of the WF10 WT virus (Fig. 2A to C), while the double mutant I100V/E101D also had a small-plaque phenotype, though less markedly so than E26K (Fig. 2C). Within the WF10 panel of viruses, the most striking visual difference was caused by the introduction of K26E, which facilitated significant larger plaque diameters (Fig. 2B and C). D316G also gave a heterogenous but significantly larger average plaque size than WT WF10. Mutations at position 26 were the only viruses to give reciprocal plaque size phenotypes in both viral backgrounds, strongly suggesting that this position is key to the phenotype.

### Residue 26 in PA modulates virus replication kinetics.

For influenza viruses, small-plaque phenotypes are often used as a marker of poor virus replication; we thus further investigated this phenotype by performing multiple cycle replication kinetics experiments with the position 26 mutants.

**FIG 3:**
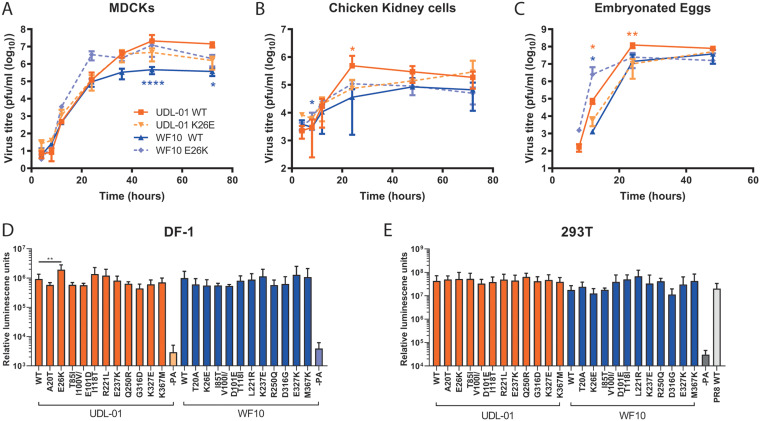
Variation at position 26 leads to differences in replication but not in polymerase activity. (A) MDCK cells and (B) CK cells were infected with the specified virus (UDL-01 WT, UDL-01 E26K, WF10 WT, or WF10 K26E) at a low multiplicity of infection (MOI) of 0.01. (C) Ten-day-old fertilized hens’ eggs infected with 100 PFU of virus. Samples were taken at the indicated time points for titer determination via plaque assay. No virus was detected prior to 8 h p.i. Data represent the average ± SD of 3 independent experiments (cells) or 5 eggs per time point. Significant differences by unpaired *t* tests (A: UDL-01 36 and 72 h p.i. and WF10 24, 36, 48, and 72 h p.i.; B: UDL-01 8, 12, 24, and 48 h p.i. and WF10 8 and 48 h p.i.; C: UDL-01 12 h p.i.) or Mann-Whitney test (A: UDL-01 4, 8, 12, 24, and 48 h p.i. and WF10 4, 8, and 12 h p.i.; B: UDL-01 4 and 72 h p.i. and WF10 4, 12, and 72 h p.i.; C: UDL-01 8, 24, and 48 h p.i. and WF10 all data points) between WT and corresponding mutant at each time point depending on distribution of data are represented with asterisks in orange (UDL-01 pair) or blue (WF10 pair). *, *P* < 0.035; **, *P* < 0.008; ****, *P* < 0.0001. (D and E) Polymerase activity of the different mutants was assessed using an influenza minireplicon assay. (D) DF-1 cells or (E) 293T were transfected with the components of the polymerase complex (PB1, PB2, PA, and NP) plus a vRNA mimic-encoding luciferase under the control of an avian RNA polymerase I promoter. For 293T cells the PB1, PB2, and NP were from PR8 to overcome the restriction of avian polymerase in these cells. At 48 h, posttransfection cells were lysed and luciferase levels measured. Data represent the mean ± SD of 3 independent experiments.

In MDCK cells infected at a low multiplicity of infection (MOI), by later time points (36 h and after), UDL-01 WT clearly grew to higher titers than those of WF10 WT, consistent with the plaque assay phenotypes (Fig. 3A). UDL-01 E26K showed slightly attenuated growth compared to UDL-01 WT, while WF10 K26E showed slightly enhanced titers compared to WT WF10, which were significantly different at the 48- and 72-h time points (Fig. 3A). Thus, PA residue 26 had significant reciprocal effects on the replication of UDL-01 and WF10 viruses in MDCK cells, recapitulating the differences seen for the MDCK plaque size phenotype.

To test if the effect of PA residue 26 amino acid substitutions held true in more biologically relevant avian systems, viral replication kinetics were assessed in primary chicken kidney (CK) cells and embryonated chicken eggs (Fig. 3B and C). In CK cells, there were consistent differences between the replication kinetics of the viruses similar to that seen in MDCKs; viruses with PA 26E (UDL-01 WT and WF10 K26E) reached peak titers at 24 h postinfection, while 26K-containing viruses replicated at a lower rate, achieving maximum titers at 48- and 72- h time points (Fig. 3B). UDL-01 E26K trended toward lower titers than UDL-01 WT, which was significant at 24 h postinfection. Likewise, WF10 K26E generally showed enhanced titers compared to those of WF10 WT; this was significant at 8 h postinfection.

In embryonated eggs, as in MDCK cells and CK cells, UDL-01 E26K showed attenuated growth compared to UDL-01 WT, significantly so at 12 and 24 h postinfection, while WF10 K26E showed enhanced growth compared to WF10 WT, significantly at 12 h postinfection (Fig. 3C). Considering these data together, we can conclude that amino acid substitutions at position 26 of PA within WF10 and UDL-01 H9N2 AIVs significantly altered the replication of the viruses in both mammalian cell lines and avian systems, indicating this attenuation is not host dependent.

### Impact of PA amino acid substitutions on polymerase activity.

As an integral part of the trimeric polymerase, influenza PA mutations have previously been shown to impact polymerase activity due to its position within the heterotrimeric polymerase complex (e.g., reference 29). To investigate the role of K26E, as well as the other mutations tested here on polymerase activity, minireplicon assays were performed in chicken DF-1 cells. Cells were transfected with expression plasmids for either the UDL-01 or WF10 polymerase components plus NP and a vRNA-reporter encoding luciferase under the control of the avian RNA polymerase I promoter. No significant differences were seen between the activities of polymerase complexes containing UDL-01 or WF10 WT polymerases (Fig. 3D). However, in contrast to the virus replication assays, UDL-01 E26K showed a small (∼2-fold) but significant increase in polymerase activity compared to that of UDL-01 WT. There were no further significant differences seen with any of the UDL-01 or WF10 mutants compared to the activity of the relevant WT control, including the reciprocal K26E change in WF10. Polymerase activity was also measured in mammalian 293T cells, but no significant differences were seen (Fig. 3E). These data suggest that the differences in plaque phenotype observed between WF10 (progenitor) and UDL-01 (reassortant) H9N2 AIVs was not unambiguously related to the ability of segment 3 to support polymerase activity alone.

### PA-E26K attenuates virus replication and pathogenicity *in vivo*.

**FIG 4:**
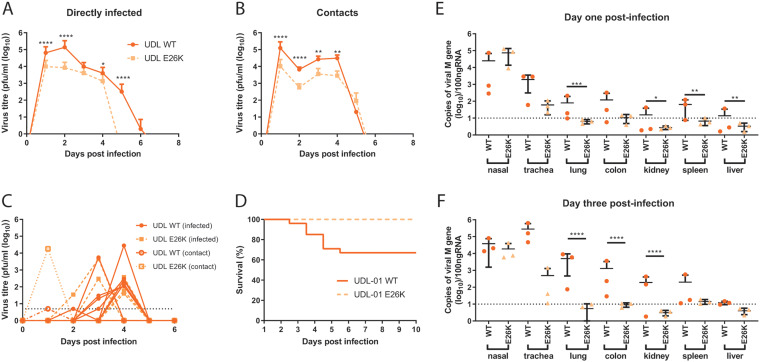
In the background of UDL-01, K26E leads to reduced shedding, mortality, and tissue tropism. Groups of 20 chickens were infected with 10^4^ PFU of either UDL-01 WT or mutant UDL-01 E26K viruses. At 1 day postinfection, 8 naive contact birds were cohoused with each group. Birds were swabbed in the buccal and cloacal cavities throughout the study duration. (A, B) Average buccal shedding profile of directly infected or contact birds and (C) individual cloacal shedding profiles of birds through duration of experiment. (D) Survival curve of birds exposed to each virus; graph includes both birds that died spontaneously or which reached a humane endpoint. (E, F) qRT-PCR for detection of the M gene of viral RNA from chicken tissues of birds culled on days 1 and 3 postinfection. A Mann-Whitney test was conducted for profiles shown in panels A and B. For profiles shown in panels E and F, an unpaired *t* test (day 1, trachea, kidney, spleen and liver; day 3, nasal, trachea, colon, kidney, spleen) or Mann-Whitney tests (day 1, lung and colon; day 3, lung and liver) between UDL-01 WT- and UDL-01 E26K-infected birds was conducted. ****, *P* < 0.001; ***, *P* < 0.0005; **, *P* < 0.0082; *, *P* < 0.033. Error bars represent ±SD of all birds swabbed (at least 4 birds/group). For survival curves in panel D, *P* < 0.0001 (log rank [Mantel-Cox] test).

The WF10-like K26E mutation appears to lead to an attenuated replication phenotype for UDL-01 *in vitro*, *ex vivo*, and *in ovo*; we therefore decided to assess the ability of UDL-01 E26K within the natural chicken host. Two groups of chickens were inoculated with either UDL-01 WT or UDL-01 E26K virus, and viral shedding, transmissibility, tissue tropism, and clinical signs were assessed. In both infected groups, all directly inoculated birds shed virus robustly into the buccal cavity, peaking early in infection (day 1 or 2) and then declining over the subsequent days (Fig. 4A). However, the UDL-01 E26K virus was shed in significantly smaller amounts and was cleared sooner; no swabs were found positive for infectious virus by day 5 in the E26K group compared to day 7 for the WT group (Fig. 4A). When the area under the shedding curve (AUC) was calculated to assess the total virus shed throughout the study period, birds directly infected with UDL-01 WT virus showed almost a 10-fold increased AUC compared to that of birds infected with the mutant UDL-01 E26K virus (215,517 versus 23,886). Therefore, the mutant UDL-01 E26K virus showed a reduced total shedding throughout the study by birds directly infected with virus.

Contact birds were introduced into each group 1 day postinoculation. All contact birds in both groups tested positive for infectious virus from the buccal cavity by 1 day postexposure, indicating robust contact transmission for both viruses (Fig. 4B). A significant reduction in buccal shedding was seen in contact birds exposed to UDL-01 E26K compared to UDL-01 WT from day 1 to day 4 postexposure; however, the delayed clearance of the mutant virus was not seen in the contact bird group, with both groups of birds clearing virus by day 6 postexposure and shedding similar levels of virus on day five postexposure (Fig. 4B). When the AUC was calculated to assess the total virus shed throughout the study period, contact birds infected with UDL-01 WT virus again showed around a 10-fold greater AUC compared to that of birds infected with the mutant UDL-01 E26K virus (187,915 versus 17,367).

Cloacal swabs from directly infected and contact birds were also analyzed; shedding was sporadic, with not all birds yielding detectable infectious virus (Fig. 4C). In total, six birds directly infected with UDL-01 WT and five with E26K shed detectable virus, along with a single contact bird from each group. More birds shed virus on consecutive days in the WT group than in the E26K group (4 birds versus 1). This sporadic and low-level virus shedding is commonly seen for some AIV subtypes, including the UDL-01 virus (30–33).

Clinical signs throughout the study were generally mild, and the majority of birds showed diarrhea with listlessness, as expected from previous reports of H9N2 infection, including UDL-01 H9N2 (30, 33). However, between days 3 and 6 postinoculation, 33% of birds (30% directly infected and 37.5% of contact birds) within the UDL-01 WT-infected group died (either spontaneously or due to reaching humane end points and being culled), despite UDL-01 being classified as a low-pathogenicity AIV (LPAIV) (Fig. 4D); analysis of these survival curves showed a statistically significant difference (*P* < 0.0001). UDL-01 has previously shown to cause high levels of morbidity, as well as occasionally low levels of mortality, in experimentally infected animals of certain chicken lines (30, 33). Birds infected with mutant UDL-01 E26K showed no mortality, indicating that this single mutation clearly attenuates the virus for pathogenicity and mortality.

### Tropism of virus in infected chickens.

To determine whether the UDL-01 E26K mutation lead to any alteration in tropism, tissues were taken from directly infected birds on days 1 and 3 postinoculation. RNA extracted from tissue samples was used for reverse transcription-quantitative PCRs (qRT-PCRs) to detect the viral M gene vRNA as a marker for presence of virus within tissues, which we have previously shown correlates well with tissue infectious virus titers (30). At both time points, M gene copy number was highest in the nasal and tracheal tissues but was also readily detectable within the lung, colon, kidney, and spleen and intermittently detected in the liver (at least within UDL-01 WT-infected birds; Fig. 4E and F). Overall, levels of RNA copies were variable between days and between different birds, but levels were highest on day 3 postinoculation, particularly within the visceral organs. Within these animals, UDL-01 WT virus was consistently present at higher levels in a number of tissues compared to birds infected with the mutant UDL-01 E26K virus. On day 1 postinoculation there were significantly higher levels of RNA within the lung, kidney, spleen, and liver of the UDL-01 WT, compared to UDL-01 E26K-infected birds (Fig. 4E). On day 3, UDL-01 E26K mutant virus was mostly undetectable in the visceral organs but detectable within the nasal tissue where levels remained high (10^4^ to 10^5^ copies of the viral M gene). The lung, colon, and kidneys also showed significantly higher levels of UDL-01 WT RNA compared to those in the mutant UDL-01 E26K (Fig. 4F). This suggested that although the UDL-01 E26K virus was able replicate efficiently in the upper respiratory tract, it was less able to disseminate through the bird and was more rapidly cleared. The expanded tissue tropism of the UDL-01 WT virus likely explains the production of some mortality in this experimental study.

### Polymorphisms at position 26 affect the host shutoff activity of the accessory protein PA-X.

As we observed little or no difference in polymerase activity with the reciprocal mutants at position 26, we hypothesized that the difference in replication could be due to WF10 having poor PA-X activity. To test this, a previously described β-galactosidase (β-gal) reporter assay was used to test the ability of the PA-X proteins from these viruses to cause host cell shutoff (19, 34). Briefly, cells were cotransfected with expression plasmids containing the different segment 3s along with a β-gal reporter plasmid, followed by enzymatic readout of β-gal activity to give a measure of host gene expression in the transfected cells and thus the ability of the different segment 3 plasmids to cause host shutoff. Previous work has suggested that the majority of influenza host cell shutoff comes from expression of PA-X rather than that of PA (19, 34, 35).

**FIG 5:**
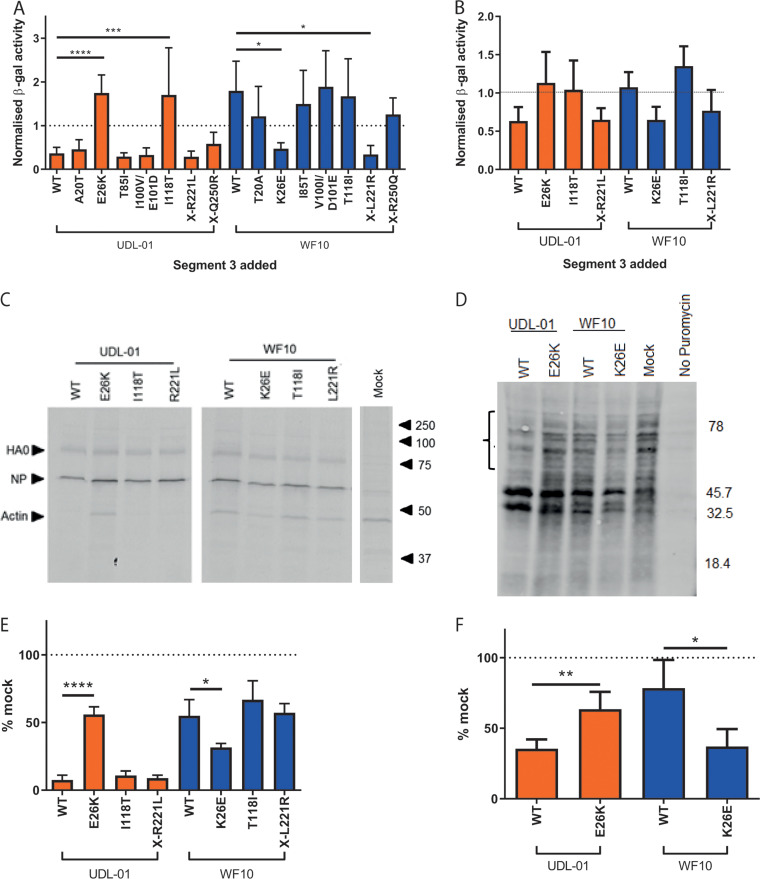
Lysine at position 26 correlates with a lack of host shutoff activity in PA-X. WT or mutant segment 3 plasmids were cotransfected into (A) 293T cells or (B) DF-1 cells with a β-gal reporter plasmid. At 48 h posttransfection, cells were lysed and levels of β-gal assessed by colorimetric enzyme assay. Results were normalized to a sample where the PA plasmid was replaced with the empty vector control. Graph represents the average of 3 independent experiments ± SD. ****, *P* < 0.0001; ***, *P* < 0.0003; *, *P* < 0.003. (A, B) One-way analysis of variance (ANOVA) with multiple comparisons (all 293T and WF10 panel in DF-1) or Kruskal-Wallis with multiple comparisons (UDL-01 panel in DF-1). (C, D) Primary chicken embryonic fibroblasts (CEF) were infected with a high MOI (3) of virus. At 7 h postinfection, cells were pulsed with ^35^S methionine for 1 h, then lysed, and proteins were separated by SDS-PAGE. Radiolabeled proteins were detected by autoradiography. (C) Representative SDS-PAGE gel with specific proteins and the positions of molecular mass (kDa) markers indicated. (D) Levels of radiolabeled actin were quantified by densitometry using ImageJ analysis software. Graph represents the average of 3 independent experiments ±SD. *, *P* = 0.02; ****, *P* < 0.0001 (one-way ANOVA with multiple comparisons). (E, F) MDCK cells were infected with a high MOI (10) of each virus. 7.5 h postinfection cells were pulsed with puromycin for 30 min. Cells were lysed, run on SDS-PAGE gels, and Western blotted for puromycin. (E) Representative Western blot gel probed for puromycin. (F) The bracket in panel E covering the areas above 45.7 kDa indicates the region quantified using ImageJ analysis software to measure the area under the curve following densitometry of this region. Data were converted to a percentage of the value seen in mock-infected cells. Graph represents average ±SD of 3 independent experiments. *, *P* = 0.0124; **, *P* = 0.007 (unpaired *t* test).

Mammalian 293T cells were transfected with plasmids with or without mutations in the shared PA/PA-X endo domain or PA-X X-ORF and β-gal activity was measured. All data were normalized to a control where segment 3 was substituted for an empty vector. UDL-01 WT segment 3 significantly reduced levels of β-gal compared to those in the empty vector control, indicating robust shutoff activity (Fig. 5A), as shown previously (34). In contrast, WF10 WT segment 3 displayed no detectable shutoff, giving equivalent β-gal signal to that of the empty vector control. When the reciprocal mutants were tested, only mutations at position 26 had a reciprocal effect, significantly removing shutoff activity in a UDL-01 background and causing shutoff activity in the WF10 background. In the UDL-01 background, I118T additionally showed significantly reduced shutoff activity, but a reciprocal effect was not seen in WF10. In the WF10 background, the X-ORF mutation X-L221R also significantly increased host shutoff activity. All other mutations showed the same phenotypes as those of their respective WT segment 3s.

The host shutoff assay was also performed in avian DF-1 cells for the mutants at positions 26, 118, and X-221 (Fig. 5B). Although not significant, the change at position 26 trended toward switching the two segment 3 phenotypes—removing shutoff activity from UDL-01 and introducing the activity into WF10 segment 3. Again, UDL-01 I118T removed shutoff activity and WF10 X-L221R partially introduced shutoff activity, but as in 293Ts, these effects were not seen reciprocally.

To assess shutoff activity further, in the context of viral infection rather than transfection and overexpression, we performed both radioactive and nonradioactive metabolic labeling experiments. To test the shutoff activity in avian cells, primary chicken embryonic fibroblast (CEF) cells were infected with a high MOI of virus containing mutations in segment 3 and were subsequently pulsed with ^35^S methionine, then lysed and run on an SDS-PAGE gel. Autoradiography was performed, and densitometry was used to measure the abundance of the highly abundant host protein actin. In accordance with the reporter assays, UDL-01 WT virus showed efficient host shutoff, with <10% of the levels of actin expressed in the mock-infected cells, whereas WF10 WT virus resulted in poor shutoff activity (>50% actin expressed versus mock; Fig. 5C and D). Reciprocal mutants at position 26 were the only mutants tested that showed any significant effect; UDL-01 E26K showed significantly poorer host shutoff, while WF10 K26E showed significantly more robust shutoff. Finally, a similar experiment was performed in MDCK cells using the nonradioactive method of puromycin pulsing and looking for levels of puromycinylated proteins in cell lysates (36). MDCKs were either infected with WT viruses or the position 26 mutants. Levels of puromycinylated products were then detected by Western blot analysis and quantified in the ∼50- to 80-kDa range, where no novel products likely to correspond to viral polypeptides were visible. As with the previous assays, UDL-01 WT gave robust shutoff, whereas WF10 WT gave poor shutoff, which could be switched significantly upon the introduction of the reciprocal mutations at position 26 (Fig. 5E and F).

Overall, these results show that UDL-01 has a PA-X capable of causing robust host shutoff in avian and mammalian cells, while WF10 does not, and the reason for this difference maps to the identity of the amino acid at position 26.

### Differences in PA-X alone are not responsible for the attenuation of WF10 compared to UDL-01.

The changes at PA position 26 are responsible for attenuation of WF10 *in vitro* and *in vivo*; these correlated better with the *in vitro* host cell shutoff activity of PA-X than with polymerase activity. To investigate whether the E26K polymorphism exerted its *in vivo* phenotypic effect via PA-X rather than through PA, we introduced a well-characterized set of nucleotide substitutions into the frameshift site (FS mutant) of PA, which has previously been shown to inhibit expression of PA-X (19, 34, 35). We further combined this FS mutation with the reciprocal mutants at position 26.

**FIG 6:**
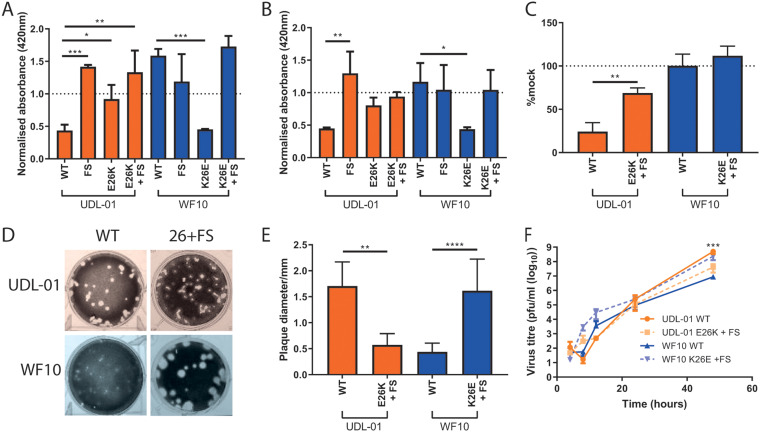
The attenuated replication of WF10 is independent of PA-X expression. H9N2 segment 3s with differing mutations were transfected into (A) 293T cells or (B) DF-1 cells along with a β-galactosidase (β-gal) reporter plasmid. At 48 h posttransfection, cells were lysed and then levels of β-gal assessed by colorimetric enzyme assay. Results were normalized to an empty vector control where no shutoff host gene expression was expected. Graph represents the average of 3 independent experiments ±SD. ***, *P* = 0.0001; **, *P* < 0.0097; *, *P* < 0.045 (one-way ANOVA [293T all and DF-1 WF10 panel] or Kruskal Wallis [DF-1 UDL-01 panel] with multiple comparisons). (C) Host cell shutoff within viral infection was then assessed, and MDCK cells were infected with each virus at a high MOI (5). At 7 h postinfection, cells were pulsed with puromycin for 30 min. Cells were lysed in SDS-PAGE buffer and Western blotted for puromycin. The area under the curve for each section was calculated and then compared to that for the mock-infected sample. The graph displays average ±SD of 3 independent experiments. **, *P* = 0.0042 (unpaired *t* test). (D and E) The plaque phenotype of viruses containing both polymorphisms at position 26 and a PA-X frameshift mutation was assessed. Viral plaque phenotypes were assessed in MDCK cells under 0.6% agarose. At 48 h postinfection, cells were fixed and stained with 0.1% crystal violet solution and (D) plaques imaged. (E) ImageJ analysis software was used to measure the diameters of 20 plaques per virus. Graph represents average ±SD. **, *P* = 0.0015; ****, *P* < 0.0001 (unpaired *t* test). (F) MDCK cells were infected with a low MOI of virus (0.01), cell supernatants were harvested at various time points postinfection and viral titers determined via plaque assay. Graph represents the average of 3 independent experiments ±SD. ***, *P* = 0.006 (unpaired *t* test [UDL-01 4 and 24 h p.i. and WF10 8, 12, 24, and 48 h p.i.] or Mann-Whitney [UDL-01 8, 12, and 48 h p.i. and WF10 4 h p.i.]). ***, between WF10 WT and WF10 K26E +FS.

We initially investigated the combined effect of position 26 and FS mutations on host shutoff in mammalian and avian cells (Fig. 6A and B). For UDL-01 WT, introduction of the E26K and/or FS mutation (individually or together) ablated shutoff activity, indicating that, as we and others have shown, segment 3 shutoff activity maps to PA-X (19, 34, 35, 37, 38). Conversely, as WF10 WT segment 3 had no shutoff activity, the FS mutant alone had no additional effect but ablated the increased shutoff activity seen when combined with WF10 K26E. An identical outcome of the mutations was observed in avian cells (Fig. 6B). Finally, using whole viruses with both mutations at position 26 and FS combined, we showed that when PA-X expression was ablated in both viruses, shutoff activity (as assayed by puromycin incorporation) was lost (Fig. 6C). Overall, these data indicate that the shutoff activity of segment 3s that contain PA 26E is entirely dependent on PA-X expression.

We next tested whether the difference in plaque and replication phenotype also mapped to PA-X and whether the differences seen previously in this study were sensitive to removal of PA-X expression. Looking at the plaque phenotypes of these combined mutants, we saw that the frameshift mutant had little or no effect on the reciprocal plaque sizes seen in the viruses; although the UDL-01 E26K plus FS mutant had a significantly smaller plaque size than that of the UDL-01 WT, the WF10 K26E plus FS virus retained its large plaque size, despite a lack of PA-X expression and shutoff activity (Fig. 6D and E). Furthermore, the replication kinetics of the combined mutant viruses exhibited a similar phenotype—WF10 K26E plus FS grew to higher titers than WF10 WT, indicating again that the enhanced replication conferred by the PA K26E mutation was independent of PA-X expression and shutoff activity (Fig. 6F). Overall, this implies that the difference in virus replication, and potentially that in pathogenicity, seen in viruses with differences at segment 3 position 26 may partially or fully map to PA rather than to PA-X alone.

## DISCUSSION

In this study, we investigated how differences in the PA gene of a progenitor (WF10) and a contemporary reassortant (UDL-01) H9N2 virus led to differences in replicative fitness. We mapped these differences to a single amino acid change of K to E at PA residue 26, which is within the endonuclease domain. Although changes at this residue did not affect virus polymerase activity, they did cause reciprocal differences in replicative fitness in both mammalian and avian systems, in cell lines, primary cells, and embryonated eggs, as well as *in vivo*, in chickens. We found that although these mutations strongly affected the shutoff activity of the accessory protein PA-X, this did not explain the differences in *in vitro* virus replication phenotype, indicating that it is likely that differences in PA function are partially or fully responsible for this.

The influenza virus accessory protein, PA-X, has been described in multiple studies as a virulence factor in avian influenza viruses (including H9N2 viruses) that can affect disease outcome in mammals or birds (22, 24, 26, 34), although other studies have found that PA-X expression can lead to an attenuated phenotype, particularly in highly pathogenic H5N1 viruses (23, 25). We found that differences between UDL-01 and WF10 at position 26 do modulate PA-X shutoff activity; however, PA-X activity alone was not responsible for the different replication phenotypes seen in these viruses. It is possible that PA-X activity may still be contributing *in vivo* but that this effect is overshadowed by a dominant PA-specific replication effect. Although several further gene products are described as being generated from influenza A virus segment 3 (for example PA-N155 and PA-N182), these products do not share the PA endo domain and therefore are unlikely to explain the difference in phenotype between UDL-01 and WF10 (39).

The poor replication and small-plaque phenotype of WF10 has been previously described by Wan and colleagues; the authors showed that the small-plaque phenotype of WF10 could be overcome by supplying the virus with the internal genes of a human H3N2 virus (40). In our study, we further map these phenotypes to a single polymorphism in the PA gene at position 26. In a separate study by Obadan and colleagues, a WF10 mutant virus library with heterogeneity in the hemagglutinin receptor binding site was used to infect quails. It was found that PA-K26E, the UDL-01-like mutation, was consistently found to spontaneously arise—further suggesting that PA-K26 is responsible for the attenuated phenotype seen in WF10, both *in vitro* and *in vivo* (41).

Throughout this study, it has been shown that PA residue 26 is responsible for the attenuated phenotype seen in WF10. When the relative distribution of polymorphisms at position 26 is looked at in the population of H9N2 viruses, or throughout avian influenza viruses in general, it becomes clear that the WF10-like K26 is very rare, with only a few viruses sharing any kind of polymorphism at this position. Over 99% of avian influenza viruses, including strains of the H5, H7, or H9 subtypes, contain the UDL-01-like E26 at this position, with a very few viruses containing lysine, glycine, aspartic acid, or glutamine (Table 2). Ultimately, the disease outcome of H9N2 infections in chickens is dependent on several factors. The presence of coinfecting bacteria or viruses can have an overriding impact on pathogenic outcome that exaggerates morbidity and mortality associated with H9N2 infection (42, 43). Likewise, the composition of the remaining influenza genes of H9N2 viruses can have a major influence on diseases outcome, outside the effect of PA 26 studied here (44). The observation that specific pathogen-free (SPF) chickens experimentally infected with H9N2 suffer less mortality and morbidity compared to naturally infected farmed chickens can perhaps partly be explained by these cofactors. Based on the data presented here, the apparent abundance of PA 26E in sequenced H9N2 viruses, and the propensity for the PA K26E substitution to occur in infected birds (41), detection of PA 26E in circulating closely related G1 lineage viruses should be associated with adaptation of H9N2 to gallinaceous poultry.

**TABLE 2:** Prevalence of PA residue 26 polymorphisms in avian influenza viruses

Virus(es)	Glutamic acid (%)	Lysine (%)	Other (% [polymorphism])
All avian influenza viruses	99.92	0.02	0.04 (glycine), 0.01 (aspartic acid), 0.01 (glutamine)
H5Nx	99.73	0.03	0.07 (glycine), 0.11 (aspartic acid), 0.03 (glutamine)
H7Nx	99.92	0.00	0.08 (glycine)
H9Nx	99.87	0.13	0.00

Understanding the molecular basis of the increased fitness of avian influenza viruses, both in avian and mammalian cells, as they continue to circulate and adapt to avian hosts is key to assessing the threat these viruses pose to food systems and to the human population. In this study, we describe a single naturally occurring polymorphism in the endo domain of PA that leads to an attenuated replication and virulence phenotype. This work will help guide future surveillance efforts and may help us better understand the molecular basis of viral fitness and virulence in the avian host.

## MATERIALS AND METHODS

### Ethics statement.

All animal experiments were carried out in strict accordance with the European and United Kingdom Home Office Regulations and the Animal (Scientific Procedures) Act 1986 Amendment regulation 2012, under the authority of a United Kingdom Home Office License (project license numbers P68D44CF4 X and PPL3002952).

### Cell lines.

Madin-Darby canine kidney (MDCK) cells, human embryonic kidney (HEK) 293T cells, and chicken DF-1 cells were maintained in Dulbecco’s modified Eagle medium (DMEM; Sigma) supplemented with 10% (vol/vol) fetal bovine serum (FBS) and 100 U/ml penicillin-streptomycin (Pen/Strep) (complete DMEM). All cells were grown at 37°C and 5% CO_2_.

Primary chicken kidney (CK) cells were generated as previously described (45). Briefly, kidneys from 3-week-old specific pathogen-free (SPF) Rhode Island Red (RIR) breed birds were shredded, washed in phosphate-buffered saline (PBS), trypsinized, then filtered. Cells were resuspended in CK growth medium (Eagle’s minimum essential medium [EMEM] plus 0.6% wt/vol bovine serum albumin [BSA], 10% vol/vol tryptose phosphate broth, and 300 U/ml penicillin/streptomycin), plated, and grown at 37°C and 5% CO2.

Primary chicken embryo fibroblasts (CEFs) were generated from 10-day-old chicken embryos. Embryos were homogenized and treated with trypsin-EDTA solution. Supernatants were passed through a metal mesh filter and centrifuged to pellet cells. Cells were resuspended in CEF medium (M199, 4% [vol/vol] FBS, and 100U/ml Pen/Strep), plated and grown at 37°C and 5% CO2.

### Viruses and reverse genetics.

A pair of H9N2 viruses were used through this study, A/chicken/Pakistan/UDL-01/2008 (UDL-01) and A/Guinea Fowl/Hong Kong/WF10/1999 (WF10). Both virus reverse genetics systems were created using the bidirectional pHW2000 plasmids (46, 47). Mutant PA segments were generated by site-directed mutagenesis or Gibson assembly (NEB).

Reverse genetics viruses were generated as previously described (46). Briefly, 250 ng of each plasmid for either UDL-01 and WF-10 viruses were cotransfected into 6-well plates of 293Ts using Lipofectamine 2000. At 16 h posttransfection, medium was changed to reverse genetics medium (DMEM plus 2 mM glutamine, 100 U/ml penicillin, 100 U/ml streptomycin, 0.14% [wt/vol] BSA, and 5 μg/ml tosylsulfonyl phenylalanyl chloromethyl ketone [TPCK]-treated trypsin). Following 48 h of incubation at 37°C and 5%CO_2_, supernatants were collected and inoculated into embryonated hens’ eggs to grow virus stocks. All viruses were Sanger sequenced to confirm that no further mutation had occurred upon growth of egg stocks. The generation of LPAI RG viruses was conducted in accordance with the Pirbright Institute’s risk assessments approved by the Pirbright Institute’s Health and Safety Biosafety department. All LPAI viruses were handled in class II microbiology safety cabinets.

### Virus plaque assays.

All plaque assays were performed in MDCK cells using 0.6% agarose overlay. Cells were stained with either 0.1% crystal violet solution (20% methanol). When plaques needed to be visualized by immunofluorescence, cells were fixed with 10% neutral buffered formalin followed by permeabilization with PBS (0.2% Triton X-100), then incubated with mouse monoclonal α-NP (1:2,000; Iqbal laboratory). Primary antibody was detected with goat anti-mouse IgG 568-conjugated secondary antibody (1:10,000; LICOR) Plates were imaged using an Odyssey Clx near-infrared fluorescence imaging system (LICOR). ImageJ was used to measure and analyze plaques.

### Minireplicon assays.

DF-1 cells were seeded into 24-well plates and cotransfected with PB2, PB1, PA, and NP, along with a firefly luciferase reporter construct under an avian pol I promoter (CKpPol I Luc) at the following concentrations: 160 ng (PB2), 160 ng (PB1), 40 ng (PA), 320 ng (NP), and 160 ng (pPol I Luc). At 48 h posttransfection, cells were lysed in passive lysis buffer (Promega), and lysates were read on a Promega GloMax multidetection unit using Luciferase assay reagent II (Promega) following the manufacturer’s instructions.

### Virus replication *in vitro* and *in ovo*.

MDCK and CK cells were inoculated with virus diluted in serum-free DMEM for 1 h at 37°C at an MOI of 0.01. Cell supernatants were taken at 4, 8, 12, 24, 48 and 72 h postinfection. After 1 h of incubation with virus, cells were then washed twice to remove unbound virus, and medium was replaced with virus growth medium for CK cells (DMEM plus 2 μg/ml TPCK-treated trypsin for MDCK cells or Eagle’s minimum essential medium [EMEM], 7% bovine serum albumin, and 10% tryptose phosphate broth). Virus titers were determined by plaque assay on MDCK cells.

Embryonated hens’ eggs (VALO breed, 10 days old) were inoculated with 100 PFU of virus into the allantoic cavity. Eggs were incubated for 4 to 72 h and culled via the schedule one method of refrigeration at 4°C for a minimum of 6 h. Harvested allantoic fluid from each egg was collected and clarified by centrifugation, and virus titers were assessed by plaque assay on MDCKs.

### Virus infection, transmission, and clinical outcome *in vivo*.

Rhode Island Red (RIR) embryos were purchased from the National Avian Research Facility (University of Edinburgh; http://www.narf.ac.uk/) and housed within the Pirbright Institute biosafety unit (BSU). Embryonated eggs were incubated for 21 days and hatched at the Pirbright Institute. Prior to the commencement of the study, all birds were swabbed (in both oropharyngeal and cloacal cavities) and bled via wing prick to confirm that they were naive to the virus. All infection experiments were performed in self-contained BioFlex B50 rigid body poultry isolators (Bell Isolation Systems) at negative pressure. Twenty birds per group were directly inoculated with 10^4^ PFU of virus intranasally. Mock-infected birds were instead inoculated with sterile PBS. One day postinoculation, 8 naive contact birds were introduced into each isolator to determine virus transmission.

Throughout the experiment, birds were swabbed in the buccal and cloacal cavities (days 1 through 8, 10, and 14 postinfection). Swabs were collected into 1 ml of virus transport medium (WHO standard). Swabs were soaked in medium and vortexed for 10 s before centrifugation. Viral titers in swabs were determined by plaque assay on MDCKs.

At days 1 and 3 postinoculation, directly infected birds were euthanized, and a panel of tissues were collected and stored in RNA later at −80°C until further processing. On day 14 postinfection, all remaining birds were culled via overdose of pentobarbital or via cervical dislocation.

Birds were observed twice daily and while procedures were carried out. Birds were monitored for the presence of clinical signs of infection. Mild clinical signs expected during the study included ruffled feathers, pale comb/wattles, eye and nasal discharge, reddened eyes, snicking, and listlessness. Additional moderate clinical signs that may be expected included drooping wings, swollen heads, and sporadic diarrhea. If any signs of severe disease were identified, including labored breathing, persistent diarrhea, sitting alone, not attempting to evade capture, or paralysis and unconsciousness, then birds were euthanized via a schedule one method, and postmortem examination was carried out.

### RNA extraction and RT-PCR from chicken tissues.

Tissue (30 mg) collected in RNA later was mixed with 750 μl of TRIzol. One sterile 5-mm stainless steel bead was added per tube, and tissues were homogenized using the Retsch MM 300 Bead Mill system (20 Hz, 4 min). Chloroform (200 μl) was added per tube, and tubes were shaken vigorously and incubated for 5 min at room temperature. Samples were centrifuged (9,200 × *g*, 30 min, and 4°C), and the top aqueous phase containing total RNA was added to a new microcentrifuge tube and the remaining fluid discarded. RNA extraction was then carried out using the Qiagen RNeasy minikit following the manufacturer’s instructions.

RNA extracted from tissue samples (100 ng) was used for qRT-PCR. All qRT-PCR analysis was completed using the Superscript III Platinum one-step qRT-PCR kit (Life Technologies) following the manufacturer’s instructions for reaction set-up. Cycling conditions were as follows: (i) a 5-min hold step at 50°C, (ii) a 2-min hold step at 95°C, and (iii) 40 cycles of 3 sec at 95°C and 30 s of annealing and extension at 60°C. Cycle threshold (*C_T_*) values were obtained using 7500 software v2.3. Mean *C_T_* values were calculated from triplicate data. Within viral M segment qRT-PCR, an M segment RNA standard curve was completed alongside the samples to quantify the amount of M gene RNA within the sample from the *C_T_* value. T7 RNA polymerase-derived transcripts from UDL-01 segment 7 were used for preparation of the standard curve.

### Host shutoff assays.

β-Galactosidase (β-gal) shutoff reporter assays were performed as previously described (19). Briefly, 293T or DF-1 cells were cotransfected with expression plasmids for the influenza segment 3 and β-gal reporter. At 48 h later, cells were lysed with Reporter lysis buffer (Promega). β-Gal expression was measured using the β-galactosidase enzyme assay system (Promega). A Promega GloMax multidetection unit was employed to read absorbance at 420 nm.

For the radio-labeling shutoff activity assays using live virus, chicken embryonic fibroblast (CEFs) were infected with 7:1 (PR8: H9N2) reassortant viruses containing the described PAs at an MOI of 3. At 6 h postinfection, cells were washed and overlaid with 1 ml of methionine-and cysteine-free DMEM supplemented with 5% dialyzed fetal calf serum (FCS) and 2 mM l-glutamine to starve the cells of methionine and cysteine. At 8 h postinfection, cells were washed and overlaid with methionine- and cysteine-free DMEM (supplemented as above) including ^35^S-methionine/cysteine protein labeling mix (PerkinElmer) at 0.8 mBq/ml. Cells were incubated at 37°C in a vented box containing activated charcoal (Fisher) for 1 h. Cells were washed once with ice-cold PBS and then cells lysed in protein loading buffer for SDS-PAGE and processed via autoradiography. Gels were fixed in gel fix solution (50% methanol and 10% acetic acid) for 5 to 15 min. Fix solution was replaced for 2 more rounds of fixing. Gels were dried in a gel dryer (Bio-Rad) by heating up to 80°C for 2 to 4 h under vacuum pressure. Dried gels were placed in a sealed cassette with an X-ray film (Thermo Fisher) overnight at minimum or until the desired signal strength was achieved. X-ray films were developed using a Konica SRX-101A Xograph film processor using manufacturers’ instructions.

For the nonradioactive shutoff activity assays using live virus, MDCKs were infected with whole H9N2 virus at an MOI of 5. At 7.5 h postinfection, cells were washed and the medium changed to complete DMEM containing 10 μg/ml of puromycin dihydrochloride from Streptomyces alboniger for 30 min. Cells were washed and then lysed in protein loading buffer for SDS-PAGE and Western blotted, probing for puromycin. Puromycylated protein synthesis was quantified in the region of the gel between 45 kDa and 80 kDa.

Protein quantification following autoradiography or antipuromycin Western blot was determined by densitometry using ImageJ analysis software.

### Bioinformatics analysis.

To assess the prevalence of different polymorphisms at position 26 of PA, every amino acid sequence of full-length PA isolates from avian hosts, excluding duplicate sequences, was downloaded from the NCBI Influenza Virus Database (https://www.ncbi.nlm.nih.gov/genomes/FLU/Database/nph-select.cgi), as of 23 May 2020. Sequences were aligned using Geneious R11.1.5, and the distribution of different amino acids was recorded.

### Statistical analysis.

All statistical analysis was carried out using GraphPad Prism 6/7 software. Distribution of data was assessed prior to deciding on the statistical test to use. For statistical analysis of plaque diameter, Kruskal-Wallis with Dunn’s multiple comparisons was conducted. For comparison of *in vitro* and *in vivo* replication and polymerase activity, unpaired *t* tests or Mann-Whitney tests were conducted. Log rank Mantel-Cox tests were conducted for survival curves. One-way analysis of variance (ANOVA) with multiple comparisons or Kruskal-Wallis with multiple comparisons were conducted for β-galactosidase reporter assays.
